# Cost-effectiveness of Internet-Delivered vs In-Person Cognitive Behavioral Therapy for Children and Adolescents With Obsessive-Compulsive Disorder

**DOI:** 10.1001/jamanetworkopen.2021.18516

**Published:** 2021-07-30

**Authors:** Kristina Aspvall, Filipa Sampaio, Fabian Lenhard, Karin Melin, Lisa Norlin, Eva Serlachius, David Mataix-Cols, Erik Andersson

**Affiliations:** 1 Centre for Psychiatry Research, Department of Clinical Neuroscience, Karolinska Institutet, Sweden; 2 Stockholm Health Care Services, Region Stockholm, Stockholm, Sweden; 3 Department of Public Health and Caring Sciences, Uppsala Universitet, Uppsala, Sweden; 4 Institute Health and Care Sciences, Sahlgrenska Academy, University of Gothenburg, Gothenburg, Sweden; 5 Region Västra Götaland, Department of Child and Adolescent Psychiatry, Sahlgrenska University Hospital, Gothenburg, Sweden; 6 Division of Psychology, Department of Clinical Neuroscience, Karolinska Institutet, Stockholm, Sweden

## Abstract

**Importance:**

Therapist-guided, internet-delivered cognitive behavioral therapy is an effective treatment option for children and adolescents with obsessive-compulsive disorder, but to our knowledge, its cost-effectiveness compared with traditional in-person treatment has not been established.

**Objective:**

To evaluate the cost-effectiveness of guided internet-delivered cognitive behavioral therapy implemented within a stepped-care model compared with in-person cognitive behavioral therapy for young people with obsessive-compulsive disorder.

**Design, Setting, and Participants:**

This economic evaluation of a randomized noninferiority trial conducted at 2 specialist obsessive-compulsive disorder clinics in Sweden enrolled 152 children and adolescents aged 8 to 17 years with obsessive-compulsive disorder, mainly through clinician referrals (110 [72%]). Recruitment began October 6, 2017, and ended May 24, 2019. Follow-up ended April 14, 2020.

**Interventions:**

Participants were randomly assigned to receive either guided internet-delivered cognitive behavioral therapy or in-person cognitive behavioral therapy during a 16-week period. At the 3-month follow-up, nonresponders in both groups were offered additional in-person cognitive behavior therapy sessions.

**Main Outcomes and Measures:**

Health outcomes were treatment response rates (primary outcome), remission rates, and quality-adjusted life-years. Cost data were collected before treatment, after treatment, at 3-month follow-up, and at 6-month follow-up (primary end point) and are presented in 2020 US dollars. The differences in incremental costs and health outcomes were compared between the groups and presented from the health care professional, health care sector, and societal perspectives.

**Results:**

A total of 152 participants (94 girls [62%]; mean [SD] age, 13.4 [2.5] years) were randomized; 151 (99%) completed the trial. At the 6-month follow-up, 50 of 74 participants (68%) in the stepped-care group and 52 of 77 participants (68%) in the in-person cognitive behavioral therapy group were classified as treatment responders (odds ratio, 1.00 [95% CI, 0.51-1.98]; *P* = .99). Health economic analyses showed that the stepped-care group used fewer therapist resources than the in-person cognitive behavioral therapy group, resulting in a mean cost savings of $2104 (95% CI, $1202-$3006) per participant for the full study period of 10 months, corresponding to a relative savings of 39%. The cost savings remained largely comparable when taking wider health care sector and societal perspectives.

**Conclusions and Relevance:**

This study suggests that, for young people with obsessive-compulsive disorder, a low-cost digital intervention followed by in-person treatment for nonresponders was cost-effective compared with in-person cognitive behavior therapy alone.

## Key Points

**Question:**

Is internet-delivered cognitive behavioral therapy followed by face-to-face treatment for nonresponders a cost-effective alternative to in-person cognitive behavioral therapy alone for children and adolescents with obsessive-compulsive disorder?

**Findings:**

In this economic evaluation of a randomized clinical trial including 152 children and adolescents with obsessive-compulsive disorder, internet-delivered cognitive behavioral therapy followed by face-to-face treatment for nonresponders was associated with reduced costs compared with traditional in-person cognitive behavioral therapy alone, without impairing effectiveness.

**Meaning:**

This study suggests that a stepped-care model of cognitive behavioral therapy delivery may be cost-effective for young people with obsessive-compulsive disorder.

## Teaser

This economic evaluation assesses the cost-effectiveness of guided internet-delivered cognitive behavioral therapy implemented within a stepped-care model compared with in-person cognitive behavioral therapy for young people with obsessive-compulsive disorder.

## Introduction

Obsessive-compulsive disorder (OCD) has a typical onset during childhood or adolescence^1^ and a lifetime prevalence of 1.3%.^2^ The disorder is associated with a marked loss of quality of life^3^ and widespread functional impairment across a number of important areas, such as education and labor market participation.^4,5^ Obsessive-compulsive disorder is also associated with increased morbidity and premature mortality from both natural and unnatural causes.^6,7,8^

The recommended first-line treatment for young people with OCD is cognitive behavioral therapy (CBT).^9,10^ Cognitive behavioral therapy is highly effective in reducing OCD symptoms,^11^ is associated with no or few known adverse events,^12^ and its effects tend to be sustained long term.^13^ However, CBT for OCD is best delivered by a multidisciplinary specialist team and is therefore in short supply.^14^ To increase the availability of high-quality CBT, we developed a therapist- and parent-guided internet-delivered CBT program for children and adolescents with OCD. Families work their way through a series of interactive online CBT modules and receive asynchronous support from a clinician via a dedicated online platform. This low-intensity intervention has demonstrated efficacy and cost-effectiveness in 3 open studies^15,16,17^ and 1 randomized waitlist clinical superiority trial.^18,19,20^ In a subsequent randomized noninferiority clinical trial,^19,20^ 152 children and adolescents with OCD were randomly assigned to receive either internet-delivered CBT or in-person CBT during a 16-week period. At the 3-month follow-up, nonresponders in both groups were offered additional in-person CBT sessions. At the primary end point (6-month follow-up), there was a noninferior difference in OCD symptom severity between the groups. Because more than 50% of participants randomly assigned to receive internet-delivered CBT were classed as treatment responders and did not require additional in-person CBT, we hypothesized that the stepped-care approach would result in cost savings without impairing effectiveness. This study formally tested this hypothesis via a health economic evaluation of our noninferiority trial, as prespecified in the study protocol.^19,20^

## Methods

### Study Design

This study is a randomized clinical noninferiority trial with an embedded health economic evaluation for children and adolescents with OCD (N = 152) comparing stepped-care CBT (n = 74) with in-person CBT alone (n = 78). Recruitment began October 6, 2017, and ended May 24, 2019. Follow-up ended April 14, 2020. Blinded clinician assessments were conducted before treatment, after treatment, at 3-month follow-up, and at 6-month follow-up (primary end point). The study protocol^21^ and primary efficacy results^22^ have been published elsewhere. The study participant flow is presented in eFigure 1 in the Supplement. The study was approved by the Regional Ethical Review Board in Stockholm, Sweden, and all participants and their parents or legal guardians provided verbal and written informed consent prior to inclusion (ClinicalTrials.gov Identifier: NCT03263546). This study followed the Consolidated Health Economic Evaluation Reporting Standards (CHEERS) reporting guideline.^23^

### Participants

Participants were 152 children and adolescents recruited from 2 specialist pediatric OCD clinics in Stockholm and Gothenburg, Sweden. Families could also self-refer to the study via a dedicated study website. Eligible participants were children and adolescents who had a primary diagnosis of OCD according to the *Diagnostic and Statistical Manual of Mental Disorders* (Fifth Edition)*,*^24^ had a total score of 16 or more on the Children’s Yale-Brown Obsessive-Compulsive Scale (scores range from 0 to 40, with higher scores indicating more severe symptoms),^25^ were between 7 and 17 years of age, were able to read and write Swedish, and had daily access to a computer with an internet connection. Participants taking psychotropic medication were required to have had a stable dose during the 6 weeks prior to inclusion. Comorbid conditions were allowed, except for organic brain disorder, global learning disabilities, autism spectrum disorder, bipolar disorder, psychosis, or severe eating disorder. Patients were excluded if they had suicidal ideation, were housebound or in need of intensive or inpatient treatment, had completed a course of CBT for OCD within the last 12 months, or had ongoing psychological treatment for OCD or an anxiety disorder. Detailed information on the study sample is presented in Table 1.

**Table 1.** Sociodemographic and Clinical Characteristics of the Sample

Characteristic	Participants, No. (%)	
	Stepped-care (n = 74)	In-person CBT (n = 78)
Sex		
Girls	46 (62)	48 (62)
Boys	28 (38)	30 (39)
Age, mean (SD) [range], y	13.4 (2.6) [8-17]	13.4 (2.5) [8-17]
Educational level of parent^a^		
Primary school	2 (3)	0
Secondary school	8 (11)	8 (10)
College or university		
<2 y	8 (11)	8 (10)
≥2 y	53 (72)	60 (77)
Doctorate	3 (4)	2 (3)
Occupational status of parent^a^		
Working	68 (92)	75 (96)
Student	0	3 (4)
On sick leave	6 (8)	0
Comorbidity		
None	50 (68)	50 (64)
Depressive episode	6 (8)	12 (15)
Anxiety disorders		
Specific phobia	9 (12)	6 (8)
Social phobia	4 (5)	4 (5)
GAD	4 (5)	3 (4)
Panic disorder	1 (1)	2 (3)
Separation anxiety	1 (1)	2 (3)
Health anxiety	0	1 (1)
Tic disorder	6 (8)	8 (10)
ADHD	6 (8)	4 (5)
Eating disorder	1 (1)	0
Medication (ongoing)		
None	69 (93)	74 (95)
SSRI	2 (3)	3 (4)
Sleep hormone	2 (3)	2 (2)
Central stimulants	2 (3)	0
Antihistamine	0	0
Baseline CY-BOCS score, mean (SD) [range]^b^	22.96 (3.64) [16-32]	22.95 (3.70) [17-33]
Baseline total societal costs, mean (SD), $	3479 (3907)	3098 (3396)
Source of referral		
Clinician	54 (73)	56 (72)
Self	20 (27)	22 (28)

Abbreviations: ADHD, attention-deficit/hyperactivity disorder; CBT, cognitive behavioral therapy; CY-BOCS, Children’s Yale-Brown Obsessive-Compulsive Scale; GAD, generalized anxiety disorder; SSRI, selective serotonin-reuptake inhibitors.

^a^ The parent mainly responsible for study participation.

^b^ CY-BOCS scores range from 0 to 40, with higher scores indicating more severe symptoms.

### Interventions

#### Stepped-Care CBT

Participants randomly assigned to receive stepped-care treatment (n = 74) first received guided internet-delivered CBT during a 16-week period. There were separate versions of the program for children and adolescents; both consisted of 14 consecutive modules that focused on psychoeducation, exposure with response prevention, and relapse prevention. Parents had access to parallel modules that specifically addressed parental aspects of the treatment, such as family accommodation of symptoms. The families had contact with a designated therapist throughout the treatment period who responded to messages through the built-in message system, provided feedback on assignments, and helped the families when problems arose. Most of the therapist contact was asynchronous through the treatment platform, but additional telephone support was provided on demand. A more detailed description of the treatment can be found elsewhere.^21,22^ Participants who were assessed as being nonresponders at the 3-month follow-up^26^ were offered up to 12 sessions of in-person CBT between the 3-month follow-up and the 6-month follow-up. The additional in-person sessions focused on exposure with response prevention according to a validated in-person CBT protocol for youths with OCD,^27^ with individually tailored adaptations regarding the degree of parental involvement, session length, and need for home visits.

#### In-Person CBT

Participants randomly assigned to receive in-person CBT (n = 78) received face-to-face treatment during a 16-week period. The treatment consisted of up to 14 sessions of manualized CBT^27^ delivered by a designated therapist; the main focus was on exposure with response prevention. Adaptations based on individual needs, such as longer sessions or home visits, were made. Nonresponders at the 3-month follow-up were offered additional face-to-face treatment, as in the stepped-care CBT group. The treatment is described in detail elsewhere.^22^

### Health Economic Evaluation

The 2 treatment groups were compared regarding health care professional costs, health care sector costs, and other societal costs in association with clinical effectiveness (cost-effectiveness analyses) and quality-adjusted life-years (QALYs; cost utility analysis). As per our protocol, the primary analysis was the cost-effectiveness analysis from the health care professional perspective using responder status at the 6-month follow-up as the health outcome. We focused mainly on the perspective of the health care professional as the payer because we expected the difference in intervention costs to be the most relevant from a policy maker perspective. However, the health care sector and societal perspectives were also included based on current recommendations.^28^ The reason for using responder status as the primary health outcome measure was that it is the most clinically relevant when evaluating the treatment effect in regular health care. Furthermore, responder status had a central part in the design of the trial because it determined which participants should be offered additional in-person treatment at the 3-month follow-up.

For completion, we also conducted a secondary cost-effectiveness analysis using remitter status as the health outcome. A patient is regarded to be in remission when he or she no longer meets syndromal criteria for OCD and has no more than minimal symptoms.^26^

#### Costs

The health care professional costs were estimated by multiplying the therapist time spent on the 2 interventions by the mean hourly licensed clinical psychologist cost in Sweden (Table 2).^29^ The therapist time spent supporting families in the internet-delivered CBT treatment was recorded automatically in the platform; therapists manually recorded time spent on occasional telephone calls. The therapist time spent during in-person CBT was manually recorded by the therapist after each session and included occasional telephone calls, administration time (ie, time spent by the therapist preparing for sessions or writing notes in the electronic medical record), and travel time (ie, time spent by the therapist traveling to and from face-to-face CBT sessions outside the clinic). Detailed information on therapist time is found in the eTable in the Supplement.

**Table 2.** Unit Costs and Sources

Resource item	Unit cost, $^a^	Source
Healthcare resources (per visit)		
General practitioner	202.91	Region Stockholm
Nurse, counselor, or physiotherapist	90.18	Region Stockholm
Specialist practitioner^b^	450.22	Sweden’s municipalities and regions
Psychologist	381.24	Sweden’s municipalities and regions
Speech and language therapist	285.88	Region Stockholm
Dietician	328.71	Region Stockholm
Medication		
Medicines	Individual product prices	The Dental and Pharmaceutical Benefits Agency of Sweden
Dietary supplements	Individual product prices	Market price from Swedish pharmacy
Support and assistance		
Specialist teacher	22.81	Own estimate
Study help	50.48	Own estimate
Support from family and friends	17.06	Estimated as cost of leisure time
Personal assistant	34.30	Swedish Insurance Agency
Support family	76.88	Sweden’s municipalities and regions
Productivity losses		
Cost per child/d at school	82.74	Own estimate based on Swedish National Agency for Education
Average wage/h in Sweden^c^	34.93	Statistics Sweden
Cost of leisure time/h	17.06	Posttax wage/h in Sweden^29^
Intervention cost		
Psychologist/h	381.24	Sweden’s municipalities and regions

^a^ All costs uprated to 2020 US dollars.

^b^ Based on a mean of 9 medical specialties.

^c^ Includes social fees of 43.3%.

For the health care sector and societal perspectives, other costs captured by the adapted parent-rated questionnaire Trimbos-iMTA questionnaire for costs associated with psychiatric illness (TiC-P)^30^ were added to the intervention costs. For the health care sector perspective, costs for other direct health care resources and medications were included. For the societal perspective, costs for social support and assistance, productivity losses due to school absenteeism and presenteeism (being physically present in class but with markedly reduced productivity because of OCD symptoms) for children, and productivity losses due to absence from paid and unpaid work (for parents) were also included. For both these perspectives, costs were estimated by multiplying resource use frequencies by unit costs (eMethods in the Supplement; Table 2^29^). The TiC-P questionnaire asked about resource use for the preceding 3 months and was completed by the parents via the online platform before treatment, after treatment, at 3-month follow-up, and at 6-month follow-up.

Costs were accumulated from baseline until 6-month follow-up by trial group (all randomized participants included). We estimated the cost for resources in 2019 Swedish krona and converted to 2020 US dollars according to the conversion rate $1 = SEK8.871 using purchasing power parities for the gross domestic product.^31^ No discounting was applied because the data on costs and outcomes were collected within a 1 year.

#### Health Outcomes

The cost-effectiveness analyses were conducted using responder status and remitter status as primary and secondary health outcomes, respectively. Treatment response was defined as at least a 35% reduction on the Children’s Yale-Brown Obsessive-Compulsive Scale and a Clinical Global Impression–Improvement score of 1 (very much improved) or 2 (much improved). Remission was defined as a score of 12 or less on the Children’s Yale-Brown Obsessive-Compulsive Scale and a Clinical Global Impression–Severity rating of 1 (normal, not at all ill) or 2 (borderline mentally ill).^26^

The secondary cost-utility analysis used QALYs as the health outcome. The self-rated Child Health Utility 9 Dimensions (CHU9D)^32^ questionnaire was used to measure health-related quality of life and provide utility weights, which in turn were used to estimate QALYs. The CHU9D is a generic pediatric preference-based measure that includes 9 items, each with 5 response options. Total QALYs during the trial period were calculated by using the area under the curve method.^33^

### Statistical Analysis

Between-group differences in baseline societal costs were analyzed with the Wilcoxon rank sum test. All *P* values were from 2-sided tests and results were deemed statistically significant at *P* < .05. The analysis was conducted with all participants according to randomization group. Multiple imputation by chained equations was used to account for missing data on the TiC-P and CHU9D, assuming the data were likely to be missing at random.^34^ Between-group differences in responder and remitter status were analyzed using a regression framework. Costs and QALYs were analyzed using generalized linear models (gamma family, log link for costs and gaussian family, identity link for QALYs) to estimate differences between groups to allow for the consideration of other distributions and functional forms to fit the data.^35^ Between-group differences in total costs for the health care sector perspective and the broader societal perspective were analyzed while controlling for baseline total costs, and total QALYs were analyzed while controlling for baseline CHU9D utilities.^36^

### Cost-effectiveness Analyses

As cost-effectiveness estimates of stepped-care vs in-person CBT, incremental differences between the groups in net costs and outcomes are presented from the 3 different perspectives. Cost-effectiveness planes show the uncertainty around the incremental cost and outcome estimates using nonparametric bootstrapping with 5000 iterations.^37^ All statistical analyses were conducted using Stata, versions 15.1 and 16.1 (StataCorp).

## Results

A total of 152 participants (94 girls [62%]; mean [SD] age, 13.4 [2.5] years) were randomized; 74 were randomly assigned to receive stepped-care treatment, and 78 to in-person CBT; 52 participants (34%) had at least 1 additional psychiatric comorbid condition (Table 1). Most participants (110 [72%]) were referred to the study by clinicians. More detailed information about the study participants can be found in the main outcome trial^22^ and in Table 1.

### Costs

For the first treatment step, the mean (SE) intervention cost was $2140 ($112) per participant for the internet-delivered CBT treatment and $4713 ($136) for the in-person treatment. At the 3-month follow-up, 34 of the 74 individuals (46%) in the stepped-care group were classified as nonresponders; 29 of these individuals accepted the offer of in-person CBT between the 3-month follow-up and 6-month follow-up, which rendered a mean (SE) cost of $3066 ($107) per patient for this additional treatment. In the in-person CBT group, 23 of 77 individuals (30%) were classified as nonresponders at the 3-month follow-up; 16 received more in-person treatment, for a mean (SE) additional cost of $3323 ($112) per patient. Thus, in total, the mean (SE) intervention cost for participants randomly assigned to the stepped-care group was $3343 ($200) per patient, and the corresponding mean (SE) intervention cost for participants randomly assigned to in-person CBT only was $5395 ($364) per patient. This resulted in a mean cost savings of $2104 (95% CI, $1202-$3006) per patient in the stepped-care group compared with the in-person CBT alone group for the whole treatment period, a relative cost savings of 39%.

There was no difference in mean (SD) total societal costs between the 2 groups at baseline (stepped care, $3479 [$3907]; in-person CBT, $3098 [$3396]; *P* = .88; Table 1). At the 6-month follow-up, the mean health care sector cost difference between the groups was $1688 (95% CI, $919-$2133), indicating significant cost savings for the stepped-care treatment compared with in-person CBT alone. From the societal perspective, there was a mean cost savings in the stepped-care treatment of $1748 (95% CI, $483-$2488) per patient compared with in-person CBT alone. A more detailed description of the costs during the whole trial period is presented in Table 3.

**Table 3.** Cost and Outcomes Over the Trial Period^a^

Cost category	Observed mean (SE)		Mean difference	
	Stepped-care (n = 74)	In-person CBT (n = 78)	Unadjusted	Adjusted (95% CI)^b^
**Costs**				
Before multiple imputation				
Health care visits	2580 (673)	2093 (300)	485	59 (−176 to 666)
Support and assistance	406 (121)	607 (183)	−207	5 (−74 to 733)
Medication/supplements	83 (38)	63 (15)	19	−9 (−14 to 21)
Parental unpaid productivity loss^c^	1500 (616)	1541 (441)	−41	38 (−95 to 586)
Parental paid productivity loss^d^	926 (166)	1806 (429)	−937	7 (−135 to 1184)
Child school absenteeism	1091 (221)	1361 (233)	−274	−75 (−149 to 135)
Child school presenteeism	905 (309)	826 (173)	79	47 (−97 to 697)
Total intervention costs	3343 (200)	5395 (364)	−2104	−2104 (−3006 to −1202)
Step 1	2140 (112)	4713 (136)		
Step 2	3066 (107)	3323 (112)		
Total health care costs^e^	6137 (492)	7684 (404)	−1569	−1688 (−2133 to −919)
Total societal costs^e^	10 917 (1188)	13 825 (1212)	−2955	−1723 (−2381 to −601)
After multiple imputation				
Total health care costs^e^	6081 (605)	7527 (492)	−1445	−1530 (−2076 to −588)
Total societal costs^e^	10 755 (1360)	13 592 (1199)	−2837	−1748 (−2488 to −483)
**Health outcomes**				
Responders	0.676	0.675	0.0004	0.0004 (−0.151 to 0.152)
Remitters	0.486	0.597	−0.111	−0.111 (−0.271 to 0.049)
Total QALYs (child)	0.620 (0.009)	0.636 (0.008)	−0.017	−0.029 (−0.055 to 0.006)

Abbreviations: CBT, cognitive behavioral therapy; QALYs, quality-adjusted life-years.

^a^ Costs are for the whole trial period of 10 months and are presented in 2020 US dollars.

^b^ Responders, remitters, and total intervention costs were not adjusted. Adjusted mean differences for other costs were calculated using generalized linear models adjusted for baseline societal cost. Total QALYs were adjusted for baseline Child Health Utility 9 Dimensions utilities.

^c^ Housework.

^d^ Work absenteeism.

^e^ Including intervention costs.

### Health Outcomes at the 6-Month Follow-up

At the 6-month follow-up, 50 of 74 participants (68%) in the stepped-care group and 52 of 77 participants (68%) in the in-person CBT group were classified as treatment responders (mean difference, 0.0004 [95% CI, −0.151 to 0.152]; Table 3), corresponding to an odds ratio of 1.00 (95% CI, 0.51-1.98; *P* = .99). The proportion of participants in remission was 49% in the stepped-care group (36 of 74) and 60% in the in-person CBT group (46 of 77; mean difference, −0.111 [95% CI, −0.271 to 0.049]), corresponding to an odds ratio of 0.64 (95% CI, 0.34-1.22; *P* = .17). The adjusted mean difference in QALYs was −0.029 (95% CI, −0.055 to 0.006).

### Cost-effectiveness Analyses

The bootstrapped estimates of incremental costs from the 3 perspectives and the difference in treatment response were equally distributed between the southwest and southeast quadrants of the cost-effectiveness planes, indicating comparable treatment effectiveness between the groups and indicating that the stepped-care treatment was less costly than in-person CBT alone (Figure). The cost-effectiveness planes for the secondary outcomes (remitter status and QALYs) as the health outcomes are presented in eFigure 2 in the Supplement. These analyses indicate that stepped-care treatment was less costly but also slightly less effective than in-person CBT alone.

**Figure.**
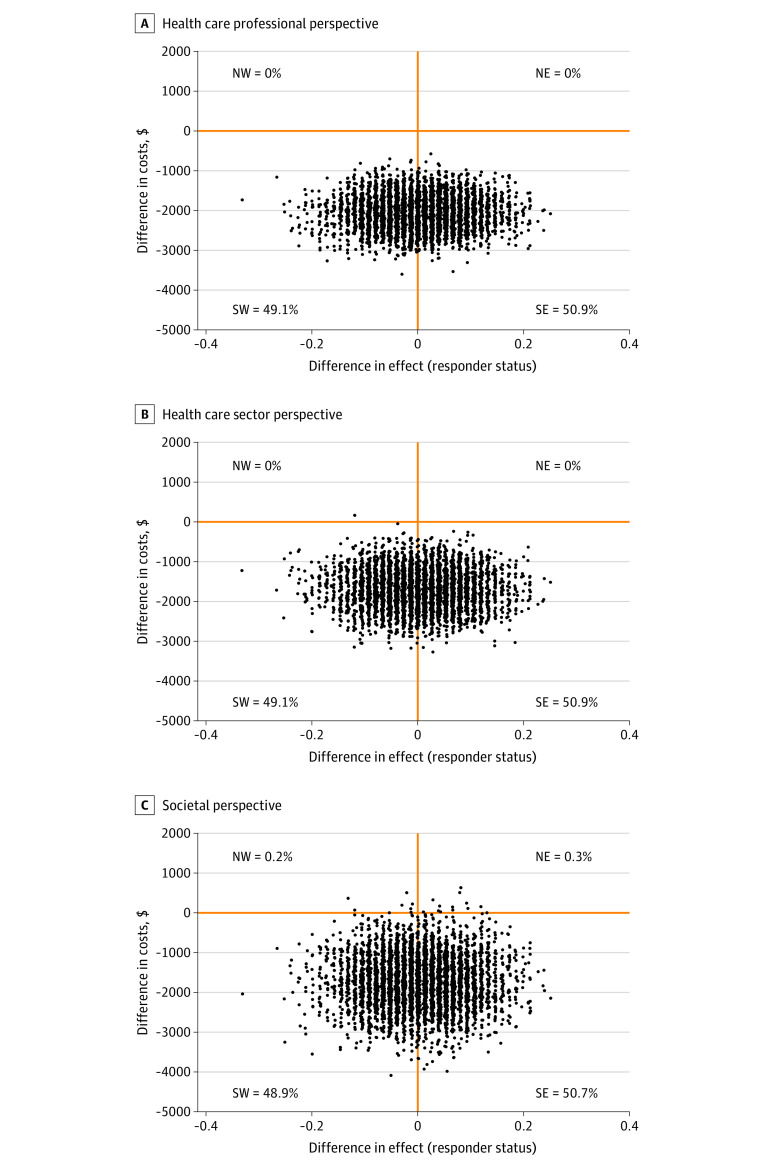
Cost-effectiveness Planes Using Responder Status as Primary Health Outcome Cost-effectiveness planes with responder status as health outcome from the health care professional perspective (A), the health care sector perspective (B), and the societal perspective (C). Each plane has 4 quadrants. Dots in the northwest (NW) quadrant indicate that stepped care is more costly and less effective. Dots in the northeast (NE) quadrant indicate that stepped care is both more costly and more effective. Dots in the southwest (SW) quadrant indicate that stepped care is both less costly and less effective. Dots in the southeast (SE) quadrant indicate that stepped care is less costly and more effective.

## Discussion

The main finding of this health economic evaluation was that delivering CBT for young people with OCD in a stepped-care fashion, with in-person CBT being reserved for nonresponders to internet-delivered CBT, was cost-effective compared with delivering in-person CBT alone. The results remained largely comparable when estimated from wider health care sector and societal perspectives.

As many as 54% of the patients (40 of 74) who received internet-delivered CBT were classified as treatment responders at the 3-month follow-up, amounting to a mean cost of only about $2140 per patient for the first treatment step. A significantly higher proportion (70% [53 of 76]) of participants randomly assigned to receive in-person CBT were classified as responders at the 3-month follow-up, but at a substantially higher mean cost of $4713 per patient for the first course of in-person CBT. Thus, it was possible to reduce the number of patients requiring in-person treatment by offering a low-cost intervention in the first step; this approach resulted in an overall cost savings of $2104 per patient for the whole study period compared with offering the standard face-to-face treatment alone. At the 6-month follow-up, the stepped-care and the in-person CBT groups had equal proportions of treatment responders (68%), but the stepped-care group had a relative cost reduction of 39%. Even when broadening the perspective to a wide-scale health care sector perspective including other health care resources and medications, and when using a societal perspective additionally including resources associated with social support and assistance as well as productivity losses (such as school and work absenteeism and presenteeism), the stepped-care approach remained a more cost-effective alternative. From a policy maker perspective, the results demonstrate that by stepwise increasing the intensity of CBT for those who do not respond sufficiently to internet-delivered CBT, it is possible to achieve the same effectiveness of treatment as that achieved with traditional in-person treatment, but at a significantly lower cost. The stepped-care approach could be even more cost-effective if treatment support were provided by less qualified clinicians (eg, trainee psychologists or other health professionals) instead of licensed clinical psychologists. Costly specialist clinician time may be better used to either treat more patients or to focus on patients with complex presentations.

Secondary analyses indicated that the stepped-care approach was less costly but that the in-person CBT group had a slightly higher proportion of remitters than the stepped care group (60% vs 49%), a statistically nonsignificant difference. It is possible that individuals receiving stepped-care treatment have a slower trajectory to remission than those randomly assigned to receive in-person CBT. Our planned 12- and 24-month follow-up analyses will hopefully shed some light on this question. The secondary cost-utility analysis also showed that the stepped-care approach produced fewer QALYs than in-person CBT. However, this between-group difference was small (the in-person CBT group scored only 0.016 points higher on the QALY outcome questionnaire than the stepped-care group, a statistically nonsignificant difference) and probably not clinically meaningful.

The only 2 previously published within-trial economic evaluations of internet-delivered CBT for OCD found that the approach was cost-effective compared with a waitlist condition for adolescents (larger proportion of responders at lower costs than the comparator)^18^ and compared with online supportive therapy for adults (incremental cost-effectiveness ratio; $931 per additional responder and $7186 per gained QALY).^38^ To our knowledge, only 1 previous study conducted a health economic evaluation of a stepped-care internet-delivered CBT model for young people with anxiety disorders.^39^ The present study is therefore, to our knowledge, a rare example of health economic evaluation of internet-delivered CBT for young people with psychiatric disorders in general.

### Strengths and Limitations

This study has some strengths. The main strengths included the high resolution of the intervention cost data, because the therapists’ time spent on each participant was meticulously recorded, and the long controlled study period, which captures both intervention costs and additional costs beyond posttreatment. Furthermore, the stepped-care treatment was evaluated against in-person CBT, which is the recommended first-line intervention for young people with OCD,^9,10^ and is thus the key comparator to guide decision makers regarding implementation. Last, most of the participants were referred by a clinician, which means that our sample was likely representative of the population typically seen in mental health clinics.

This study also has some limitations. The trial was conducted in Sweden, which has a tax-funded universal health system that may affect the interpretation of the results. The chosen scenario incorporated a full publicly funded health care system in which the focus is on lowering the costs for the health care professional. However, the stepped-care approach may result in delayed response to treatment for some individuals and may therefore not be the preferred choice for policy makers operating in other health care contexts with immediate access to high-quality specialist treatment for OCD. Future research should expand to other countries and health care contexts to evaluate the cost-effectiveness of stepped-care approaches from different payer perspectives in different samples. Another limitation was that other health care resources and societal costs were assessed retrospectively with a parent-reported measure. Although this method is considered valid^40^ and recommended in guidelines,^41^ an additional objective assessment could have strengthened the results further.

## Conclusions

This study suggests that a stepped-care model in which young people with OCD are first offered a low-cost digital CBT intervention followed by traditional in-person therapy for nonresponders is as effective as, and more cost-effective than, the traditional in-office treatment format. The findings provide important implications for clinical practice and health policy.

## Notes

Supplement. **eMethods.** Costs (Continued) **eFigure 1.** Flow Chart **eFigure 2.** Cost-effectiveness Planes Using Remitter Status and QALYs as Secondary Health Outcomes **eTable.** Therapist Time eReferences.
