# Supplementary material for: Cost-effectiveness of Internet-Delivered vs In-Person Cognitive Behavioral Therapy for Children and Adolescents With Obsessive-Compulsive Disorder
Source: JAMA Netw Open. 2021 Jul 30;4(7):e2118516. doi: 10.1001/jamanetworkopen.2021.18516 (PMC8325072; doi:10.1001/jamanetworkopen.2021.18516)
Supplement: Supplement. — eMethods. Costs (Continued) eFigure 1. Flow Chart eFigure 2. Cost-effectiveness Planes Using Remitter Status and QALYs as Secondary Health Outcomes eTable. Therapist Time eReferences. [file jamanetwopen-e2118516-s001.pdf]

Supplementary Online Content

Aspvall K, Sampaio F, Lenhard F, et al. Cost-effectiveness of internet-delivered vs in-person cognitive behavioral therapy for children and adolescents with obsessive-compulsive disorder. *JAMA Netw Open.* 2021;4(7):e2118516. doi:10.1001/jamanetworkopen.2021.18516

eMethods. Costs (continued)
eFigure 1. Flow Chart
eFigure 2. Cost-effectiveness Planes Using Remitter Status and QALYs as Secondary Health Outcomes
eTable. Therapist Time
eReferences.

This supplementary material has been provided by the authors to give readers additional information about their work.

© 2021 Aspvall K et al. *JAMA Network Open.*

eMethods. Costs (continued)

Healthcare resources used were costed using national pricelists and medications were costed using market prices. Unit costs for resources related to social support and assistance were sourced from published sources or based on authors´ own estimates. Productivity losses for both children and parents were estimated using the human capital approach.[1] Productivity losses due to absenteeism from school were estimated by multiplying the number of days not at school by the daily cost of a child in middle to high school education.[2] Productivity losses due to absenteeism from paid work were the product of the number of days off from work by parents due to the child's illness by the average hourly salary rate in Sweden including social fees.[3] Losses due to absenteeism from unpaid work were the product of the number of days not performing unpaid work by the estimated hourly cost of leisure time.[4] Productivity losses related to reduced efficiency at school were estimated in the same fashion as productivity losses due to absenteeism, but additionally multiplied by a weighed score representing how the illness impacted the participants productivity.[5]

© 2021 Aspvall K et al. *JAMA Network Open.*

eFigure. Flow Chart

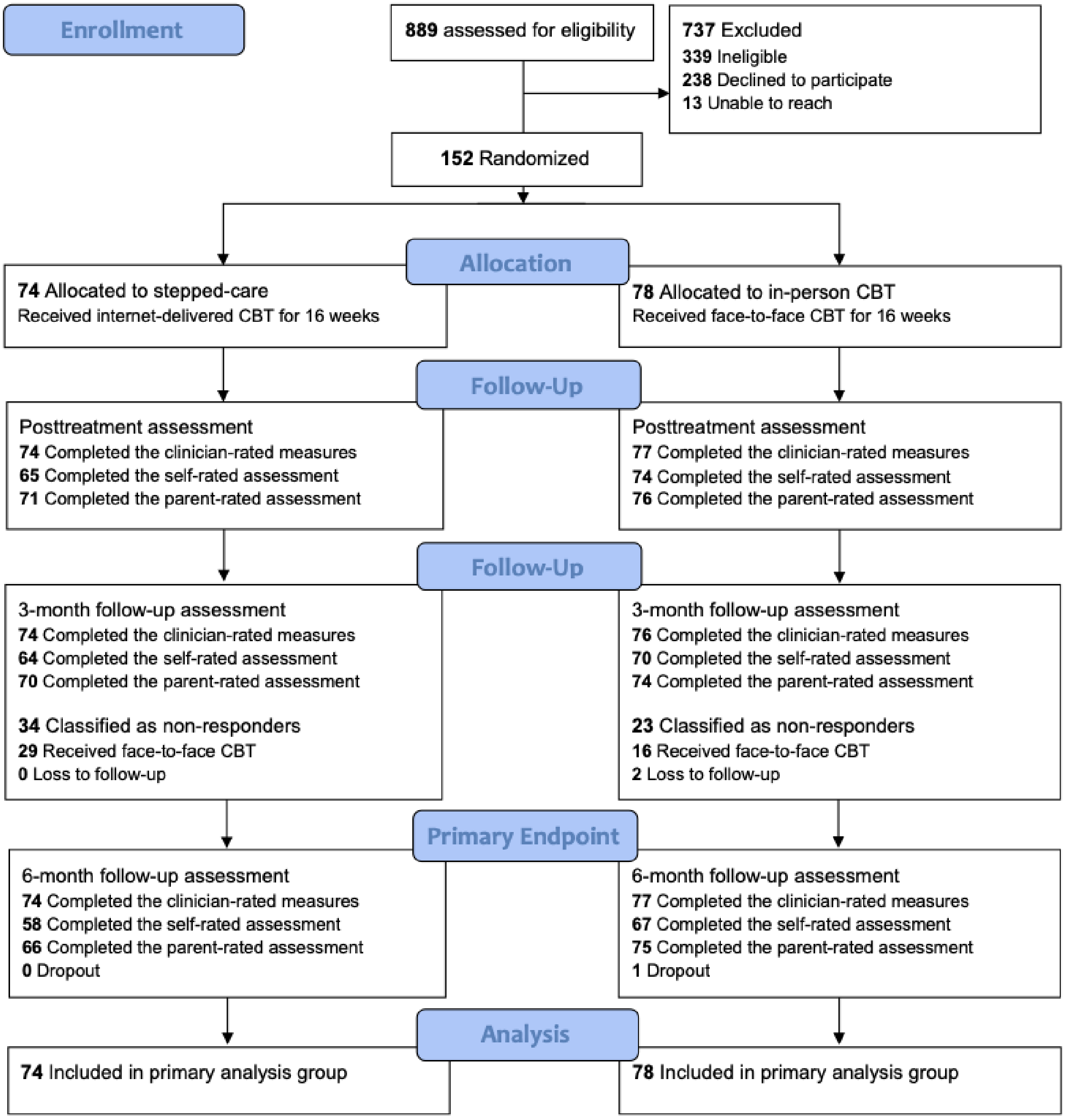

© 2021 Aspvall K et al. *JAMA Network Open.*

eFigure 2. Cost-effectiveness Planes Using Remitter Status and QALYs as Secondary Health Outcomes

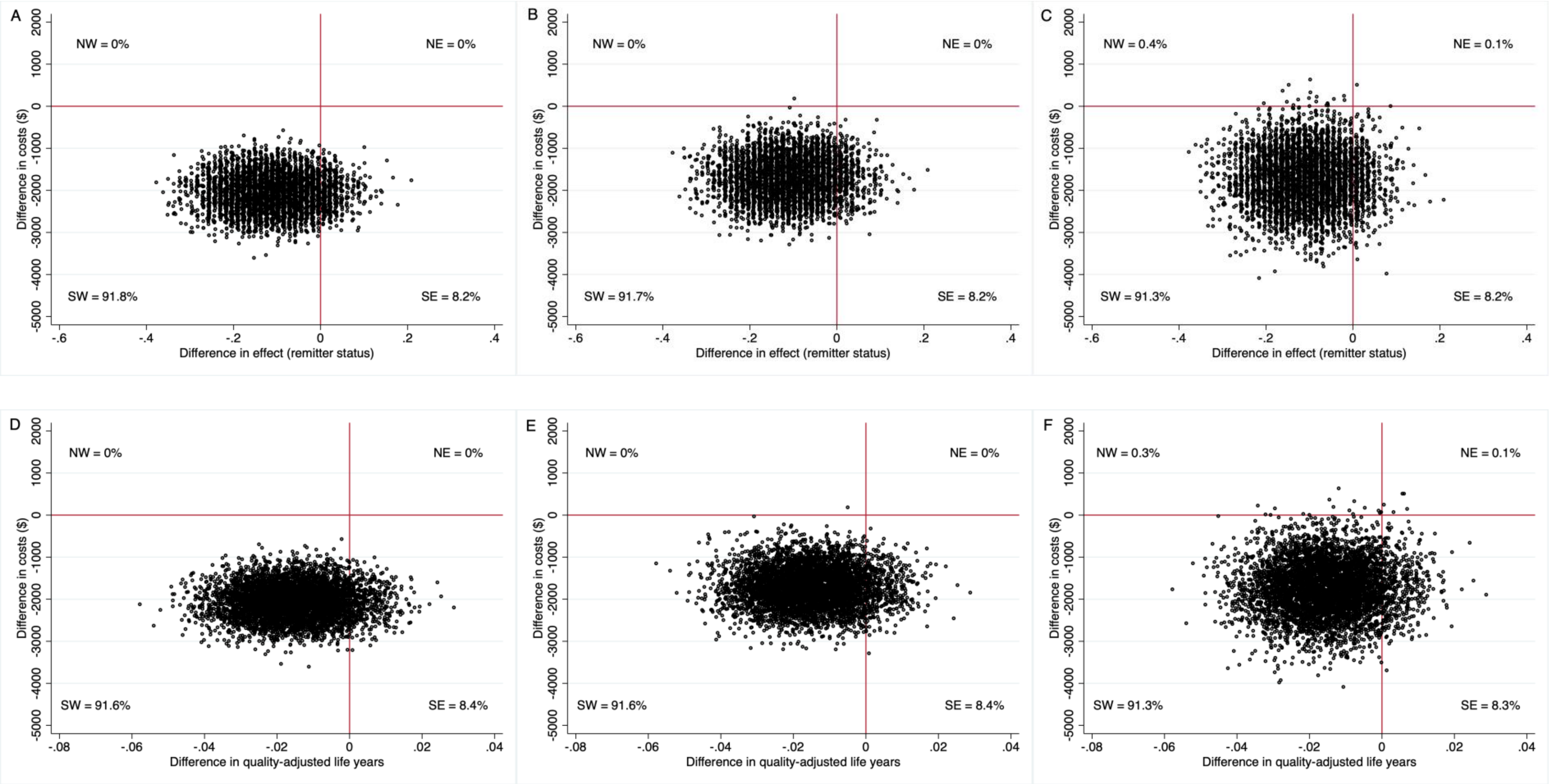

Cost-effectiveness planes with responder status (A-C) and quality-adjusted life years (QALYs; D-F) as health outcomes from the healthcare provider perspective (A and D), the healthcare sector perspective (B and E), and the societal perspective (C and F). Each plane has four quadrants. Dots in the northwest quadrant indicate that stepped-care is more costly and less effective. Dots in the northeast quadrant indicate that stepped-care is both more costly and more effective. Dots in the southwest quadrant indicate that stepped-care is both less costly and less effective. Dots in the southeast quadrant indicate that stepped-care is less costly and more effective.

© 2021 Aspvall K et al. *JAMA Network Open.*

**eTable.** Therapist Time		
	**Mean (*SD*)**	
	**Stepped-care (*n*=74)**	**In-person CBT (*n*=78)**
Step 1		
Total		
Patient contact [a]	335.00 (217.13)	577.19 (190.46)
Travel time [b]	0 (0)	11.97 (49.57)
Administration [c]	1.84 (3.83)	152.65 (90.85)
Step 2 [d]		
Total		
Patient contact [a]	390.97 (160.50)	386.56 (158.93)
Travel time [b]	8.38 (37.44)	38.44 (67.62)
Administration [c]	83.14 (33.86)	98 (39.09)
Step 1 + step 2		
Total therapist time		

[a] Patient contact was defined as time spend on correspondence in the internet-delivered CBT platform, face-to-face CBT sessions and phone calls, and includes time with both the patient and the parents.
[b] Travel time is the time spent by the therapist traveling to/from face-to-face CBT sessions when doing home visits or exposure other places than at the clinic.
[c] Administration time includes time spent by the therapist preparing for sessions or writing in the electronic medical record.
[d] Time for nonresponders who received additional in-person CBT treatment, *n*=29 in the stepped-care group and *n*=16 in the in-person CBT group.
Abbreviation: CBT, cognitive behavioral therapy.

© 2021 Aspvall K et al. *JAMA Network Open.*

eReferences.

1. Koopmanschap MA, Rutten FF. A practical guide for calculating indirect costs of disease. *Pharmacoeconomics.* 1996;10(5):460-466.
2. The Swedish National Agency for Education. Kostnader för skolväsendet och annan pedagogisk verksamhet 2017. https://www.skolverket.se/publikationer?id=3981. Published 2018. Accessed.
3. Statistics Sweden [Statistiska centralbyrån]. Lönedatabasen [Salary database]. http://www.scb.se/. Published 2019. Accessed.
4. *Cost-Effectiveness in Health and Medicine.* Second Edition ed: Oxford University Press; 2017.
5. Kigozi J, Jowett S, Lewis M, Barton P, Coast J. The Estimation and Inclusion of Presenteeism Costs in Applied Economic Evaluation: A Systematic Review. *Value Health.* 2017;20(3):496-506.

© 2021 Aspvall K et al. *JAMA Network Open.*
